# Dissatisfaction with Married Life in Men Is Related to Increased Stroke and All-Cause Mortality

**DOI:** 10.3390/jcm10081729

**Published:** 2021-04-16

**Authors:** Shahar Lev-ari, Yftach Gepner, Uri Goldbourt

**Affiliations:** 1 Department of Health Promotion, School of Public Health, Sackler Faculty of Medicine, Tel-Aviv University, Tel-Aviv 6997801, Israel; 2 Department of Epidemiology and Preventive Medicine, School of Public Health, Sackler Faculty of Medicine, Sylvan Adams Sports Institute, Tel-Aviv University, Tel-Aviv 6997801, Israel; gepner@tauex.tau.ac.il; 3 Department of Epidemiology and Preventive Medicine, School of Public Health, and the Henry N. Neufeld Cardiac Research Institute, Sackler Faculty of Medicine, Tel-Aviv University, Tel-Aviv 6997801, Israel; goldbu1@tauex.tau.ac.il

**Keywords:** marital satisfaction, all-cause mortality, cohort study

## Abstract

The objectives of this study were to assess the association between marital satisfaction and specific and all-cause mortality, and to examine whether this association is independent of other known risk factors for early mortality. In this prospective cohort, male Israeli civil servants and municipal employees (*n* = 8945) underwent an extensive appraisal of health and behavioral patterns and were followed for more than three decades. Cox proportional hazards analysis was used to estimate the relative risks for stroke and all-cause mortality over time across marital satisfaction categories. During the 32 years of follow-up, 5736 (64.1%) died. Dissatisfaction with married life was related to increased long-term risk of stroke (HR = 1.94; 95%CI, 1.41–2.90) and all-cause mortality (HR = 1.21; 95%CI, 1.04–1.41). The latter association was of a similar order of magnitude to other known risk factors for early mortality, such as people with a history of smoking (HR = 1.37; 95%CI, 1.30–1.48) compared to people who have never smoked and for physically inactive participants (HR = 1.21; 95%CI, 1.14–1.37) compared to physically active participants. The results of our study suggest that marital dissatisfaction may predict an elevated risk of all-cause mortality. Assessing marital satisfaction and measuring the health benefits of marital education programs for couples should be implemented as part of health promotion strategies for the general population.

## 1. Introduction

Marriage has been described as the most important and fundamental human relationship because it provides the primary structure for establishing a family and social support [1]. Research indicates that the quality of the marital relationship is associated with health outcomes [2,3]. Marital quality is defined through a subjective, global evaluation of the relationship and behaviors in the relationship [4]. A previous meta-analysis [5] concluded that higher marital quality was related to lower risk of mortality (mean effect size, r = 0.11). Marital satisfaction is an important factor of marital quality; other factors include cohesion, affection, and conflict. Several studies found that marital satisfaction is associated with lower risk of early mortality among populations with disease-related issues (e.g., congestive heart failure [6] and coronary artery bypass grafting [7]). However, we cannot draw conclusions from these studies regarding whether marital satisfaction can predict long-term health outcomes, such as mortality, among samples that are not health-compromised.

A recent study on a large population of American adults (18–90) found that marital dissatisfaction was significantly and negatively associated with all-cause mortality [8]. While the results of this study were adjusted for demographic variables, they did not consider health risk factors (blood pressure, cholesterol, diabetes), and lifestyle factors (physical activity, cigarette smoking) that may confound the risk for all-cause mortality. Given the scarcity of the literature on this subject, our purpose was to gain a better understanding of the relationships between marital satisfaction and long-term mortality.

The specific aims of the current analysis were to (1) assess the relationship between marital satisfaction in men and rates of all-cause mortality over four decades and (2) assess whether this association is mediated through known risk factors for stroke and all-cause mortality.

## 2. Materials and Methods

This was a follow-up study based on the Israeli Ischemic Heart Disease (IIHD) prospective cohort. The IIHD was a longitudinal investigation of risk factors for disease in Israeli subjects. This cohort included a wide range of occupations and socioeconomic levels that were present in the male working population of Israel at the time of inclusion. At the time of recruitment, subjects underwent an extensive appraisal of health and behavioral patterns, including a structured psychosocial questionnaire, and were then followed over a long period to record mortality.

### 2.1. Subjects

A sample of 8945 male civil servants and municipal employees participated in the IIHD Project in 1965. Participants were chosen by stratified sampling based on an age of 40 years and above at time of inclusion and with place of work confined to the three largest urban areas in Israel (Tel-Aviv, Jerusalem, and Haifa). The sample was designed to include members of six geographical regions (central Europe, eastern Europe, the Balkans, the Mideast, northern Africa, and Israel) in a combination approximately proportional to the general Israeli male population of a similar age at that time. 

### 2.2. Data Assessment

Participants underwent clinical and blood biochemical evaluations as previously described [9,10]. Blood pressure was measured in the right arm, with the subject in the recumbent position, 30–45 min after arrival at the clinic, and again, 15–30 min later. Cigarette smoking was recorded as six separate categories, including never smoking, smoking cigar or pipe, quitters, and three different categories according to the amount smoked. Subjects were also classified as ever-smoked or not. The participants were questioned about their diet in an effort to obtain information about their usual total food intake, with special emphasis on the type of, and amount of, fat. The quantity (usual portion) was ascertained, as was the frequency with which different foods were eaten in a day, week, or month. Food models in three sizes were on display to help the subjects estimate the size of their usual portion. Details of the dietary survey have been reported elsewhere [11].

Diabetes mellitus was diagnosed on the basis of anti-diabetic oral therapy or insulin use at baseline in addition to casual blood glucose levels. A two-hour glucose tolerance test was then carried out to determine the participant’s diabetic status. Socioeconomic status (SES) was assessed by categorizing education and occupation data into five strata, with the lowest stratum including laborers with an up-to elementary school education, and the highest stratum including men with at least some university or equivalent education, engaged in professional or teaching work. 

### 2.3. Stroke and All-Cause Mortality Assessment

The underlying cause of death was documented as a case-by-case determination by a review panel through the mid-1970s and by using the International Classification of Diseases (ICD) codes thereafter. Deaths from stroke were based on ICD-9 codes 431–438, and those from Coronary Heart Disease (CHD) by codes 410, 411, 414, and 798. For the earlier (pre-1971) deaths, a comparison of death certificates with the analyses of hospital records by the panel yielded a 90% agreement. Information on mortality from 1970 until the end of the 32-year follow-up was derived from the Israeli Mortality Registry.

### 2.4. Marital Satisfaction Assessment

Subjects completed a structured psychosocial questionnaire at baseline. This was based on previous experience and a pre-test of a more detailed questionnaire among port workers. Trained interviewers administered the questionnaire following a detailed protocol. Every subject was asked to record the degree to which they were satisfied with their marriage (marriage is perceived as 1—very successful, 2—successful, 3—not so successful, 4—unsuccessful). The advantage of this measure is that it is more sensitive to variation in dissatisfaction levels (“marriage is not so successful” vs. marriage is unsuccessful”) compared to validated single-item marital satisfaction measures used previously in large national surveys [12,13]. These had only 3 categories (1—“very happy”, 2—“moderately happy”, 3—“not too happy”, with my marriage) and were therefore less sensitive to degrees of dissatisfaction. 

In addition, participants were asked whether they had experienced or were experiencing family difficulties. These parameters have previously been described in detail [12,13] and were scored on a Likert scale (0—no problems to 4—four or more problems). Severe and very severe family problems, which were defined a priori, were scored on the family difficulties scale (i.e., serious—3 problems, very serious—4 or more problems). The outcome was recorded as a binary outcome and the results were assessed by the multivariate model. Never married subjects (2.34%) were not asked to take part in this questionnaire and were not included in further analysis related to this questionnaire. Subjects who refused to answer (6.98%) were excluded from the analysis. 

The current study is based on the IIHD study, which was a collaborative project involving the National Heart and Lung Institute, NIH, USA, the Israel Civil Service Commission, and the Hadassah Medical Organization and was conducted at the beginning of the 1960s, at a time when ethical review boards did not yet exist in Israel. However, all participants provided their oral consent to take part in the study upon their recruitment in 1963 following explanations regarding the study objectives and the long-term follow-up. In addition, the Tel Aviv University Ethical Review Board approved the linkage of the IIHD database with the Israel Population Registry. Prof. Goldbourt, an author in this publication, is legally responsible for the IIHD study database and has approved its use for the purpose of this study.

### 2.5. Statistical Analysis

An order-directed score test for trend was used to test the departure of sample results from the null hypothesis that specific or all-cause mortality is not connected to baseline marital satisfaction assessment. Survival across baseline marital satisfaction categories was estimated by the Kaplan–Meier method with right-censoring at the time of last follow-up and compared by the log-rank test. Cox proportional hazards analysis was used to estimate relative hazards and 95 percent confidence intervals for stroke and all-cause mortality over time across marital satisfaction categories. Data in the multivariate model were adjusted for age (5-year increments), hypertension, cholesterol, diabetes mellitus, leisure-time physical activity, SES, cigarette smoking, and family problems at any time up to and including 1965. The proportional hazards assumption was tested with the Schoenfeld residuals, and no violations were detected. Statistical analysis was carried out using the Stata statistical package, version 16 (Stata, College Station, TX, USA). 

## 3. Results

A total of 8945 men were included in the analysis. Baseline characteristics across categories of marital satisfaction are presented in Table 1. The average age of the population at recruitment (1965) was 49.1 ± 6.7 years. Participants with low marital satisfaction had higher levels of systolic (*p* = 0.01) and diastolic blood pressure (*p* < 0.01), lower socioeconomic status (*p* < 0.01), and higher rates of family problems (*p* < 0.01) than participants with high marital satisfaction. Other variables, including total cholesterol, body mass index (BMI), high-density lipoprotein (HDL), and diabetes mellitus, as well as dietary nutrient intake (percent of calories derived from fat, carbohydrates, and protein) were similar across all marital satisfaction categories (*p* = 0.995). About two-thirds of the participants had smoked at some time, with the number rising with decreased marital satisfaction.

**Figure 1:**
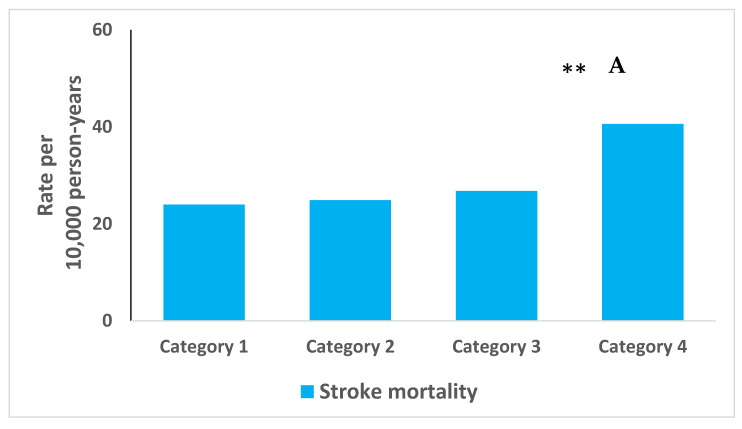

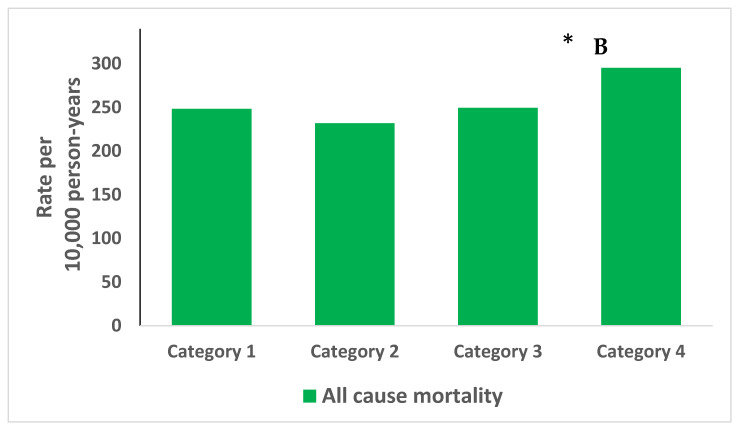
Rate per 10,000 person years mortality from stroke (**A**) or all-cause (**B**) according to category of family satisfaction. Marital satisfaction categories: marriage is perceived as 1—very successful, 2—successful, 3—not so successful, 4—unsuccessful. (* *p* = 0.036, ** *p* < 0.001).

During the 32 years of follow-up, 5736 (64.1%) subjects died, and 595 (6.7%) died from a stroke. Rates of stroke mortality increased by 69.2% from 24.0 (per 10,000 person years) in the most satisfied group to 40.6 (per 10,000 person years) in the least satisfied group (Figure 1A). Rates of all-cause mortality increased by 19% from 248.5 (per 10,000 person years) in the most satisfied group to 295.3 (per 10,000 person years) in the least satisfied group (Figure 1B). A sensitivity analysis found that the rate of mortality of younger (<50 years old) participants in the least satisfied category at recruitment was 39.4% higher than those in the most satisfied category (*p* = 0.0007). A less dramatic increase of 6.5% was seen in older (>50 years old) participants (*p* = 0.314).

**Figure 2:**
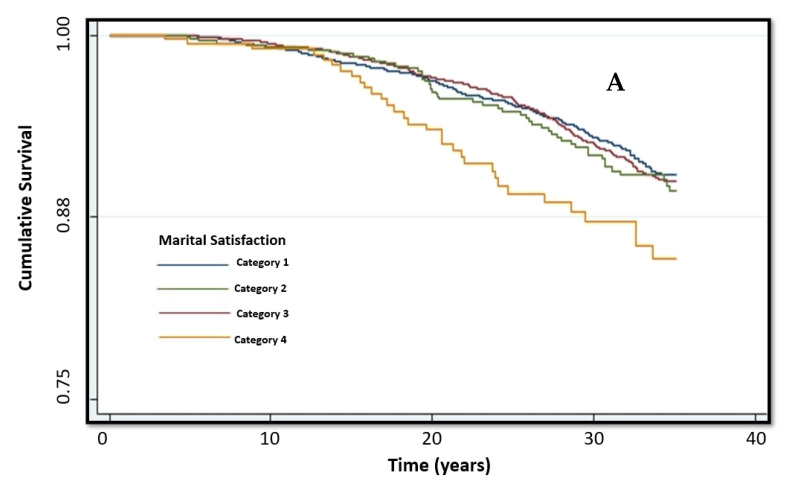

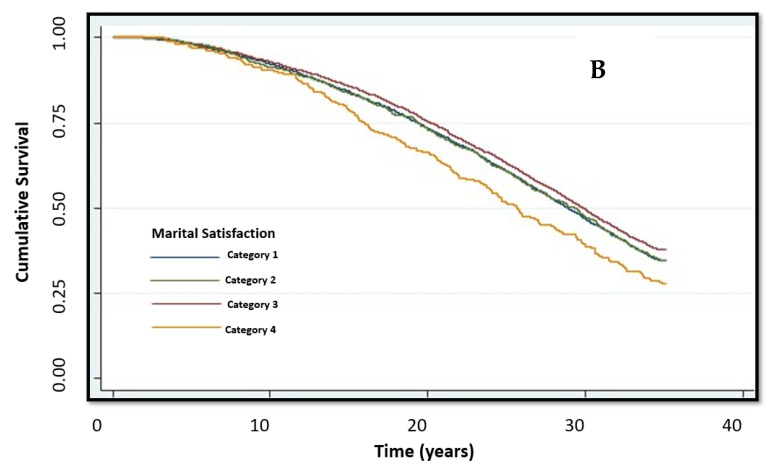
Kaplan–Meier survival curve for (**A**) stroke and (**B**) all-cause mortality. Marital satisfaction categories: marriage is perceived as 1—very successful, 2—successful, 3—not so successful, 4—unsuccessful. Participants in the lowest category of marital satisfaction had higher stroke mortality (log-rank: *p* = 0.018) and all-cause mortality (log-rank: *p* < 0.001) compared to the other marital satisfaction categories.

Kaplan–Meier survival curves for stroke and all-cause mortality as a function of marital satisfaction variability are presented in Figure 2. Participants in the lowest category of marital satisfaction had higher stroke mortality (log-rank: *p* = 0.018) and all-cause mortality (log-rank: *p* < 0.001) compared to the other marital satisfaction categories.

Multivariate analysis of 32-year mortality revealed a significant association between marital dissatisfaction and stroke and all-cause mortality (Table 2, model 1; adjusted for age). These associations persisted when the analysis was adjusted for established risk factors of mortality (Table 2, model 2; adjusted for age, ischemic heart disease, diabetes mellitus, systolic blood pressure, smoking, BMI, leisure-time physical activity, and SES), and for family problems (Table 2, model 3; model 2 further adjusted for family problems). An interaction test between marital satisfaction categories and family problem categories in putative affecting mortality yielded *p* = 0.141.

Assessments of additional lifestyle factors found that smoking (men who had ever smoked) was associated with excess risk of all-cause mortality (HR = 1.37; 95%CI, 1.30–1.48) compared to people who had never smoked. Physically inactive participants also exhibited a higher risk of all-cause mortality (HR = 1.21; 95%CI, 1.14–1.37) compared to physically active participants.

## 4. Discussion

The results of this long-term follow-up study of male civil servants and municipal employees indicate that low marital satisfaction exposes working men to an increased long-term risk of all-cause mortality, and that this association is of a similar order of magnitude to that of other known risk factors (smoking and leisure-time physical activity) for all-cause mortality.

Our results support previous reports that marital satisfaction is associated with physical health outcomes [2] and extends their findings by demonstrating that marital dissatisfaction, as assessed over more than 32 years of follow-up, is an important determinant of increased long-term risk for stroke and all-cause mortality. Dissatisfaction of men with married life was clearly related to an increase in the long-term risk of stroke (HR = 1.94; 95%CI, 1.41–2.90) and all-cause mortality (HR = 1.21, CI 1.04–1.41). This relationship may be mediated through multiple mechanisms: positive (e.g., physical exercise) and negative (e.g., cigarette smoking) health behaviors [14], psychosocial characteristics (e.g., depression, social support) [15], socioeconomic status [16], and physical health status (e.g., blood pressure). Our findings demonstrate that the association persisted even when the analysis was adjusted for established risk factors of mortality (ischemic heart disease, diabetes mellitus, systolic blood pressure and smoking, leisure-time physical activity, SES, and family problems). This relationship between marital satisfaction and mortality was comparable to that of smokers (HR = 1.37; 95%CI, 1.30–1.48) compared to never smokers and that of physically inactive (HR = 1.21; 95%CI, 1.14–1.37) compared to physically active participants. There has been extensive research into lifestyle factors such as smoking [17,18,19] and physical inactivity [20,21,22] as risk factors for premature mortality. Our study suggests that marital dissatisfaction is an equally important health factor for consideration as a risk for specific and all-cause mortality. 

Although it is a highly desirable state, achieving marital satisfaction is not easy [23,24]. Sensitivity analysis revealed that low marital satisfaction in younger participants (<50 years old) was more closely associated with a major increase in risk of stroke than in older (>50 years old) participants. A number of studies have reported that most divorces occur in younger couples [25,26] and that marital dissolution is linked to early mortality [27,28]. Developmental and accommodation theories have proposed that conflict, emotional behaviors, and dissatisfaction may diminish over time as couples become more tolerant of one another [29,30]. There have also been suggestions that educating and training young couples on positive psychology techniques, communication skills, and parenting strategies may be beneficial for developing family resilience and enhancing marital satisfaction [31,32,33,34]. These techniques may be usefully implemented as part of health promotion strategies designed for the general population. 

Our results demonstrate a strong link between family difficulties and marital satisfaction. In the group with the highest marital satisfaction rates, only 1.3% reported serious or very serious family problems compared to a value of 30.4% in the group with the lowest marital satisfaction rates. We have previously shown that family problems are associated with higher mortality rates of stroke [35]. However, here, our multivariate analysis revealed that the association between marital satisfaction and stroke or all-cause mortality was independent of the family problems variable. This may indicate that a single assessment of a self-reported attitude towards one’s own marriage may predict a higher risk of mortality. Accordingly, marital satisfaction assessment may be a valuable component in national health surveys. 

Strengths of the study include the large representative sample of 8945 participants, the long-term follow-up, and the analysis of subjective (self-reported) and objective confounders. However, there are also some limitations: Marital satisfaction was assessed at only one time-point. Therefore, we were unable to control for changes in marital satisfaction or marital status (e.g., separation or divorce), which are known to be linked to mortality [27]. Although some studies show that marital quality is fairly stable over time, particularly after the early years of marriage [36,37], repeated assessments of marital satisfaction and marital status would provide a stronger association between marital satisfaction and mortality. Secondly, only male participants were recruited in this study, precluding the assessment of marital satisfaction and mortality in women. A previous study on a US population reported that gender did not moderate the association between marital satisfaction and all-cause mortality [8]. Interestingly, marital satisfaction may affect the longevity of the married partner. A dyadic survival analysis using a representative sample of 4374 elderly couples followed for up to eight years showed that having a happy spouse was associated with a significantly lower risk of mortality (one standard deviation higher level of spousal life satisfaction was associated with a 13% lower mortality risk within the following eight years, CI = 0.83–0.91, *p* < 0.001; [38]). Finally, while we did assess family problems, we did not include other variables that could confound or mediate the relationship between marital satisfaction and stroke and all-cause mortality. Dissatisfaction with married life, beyond its individual importance and potential effect, may also serve as a proxy for other, unmeasured psychosocial characteristics, such as depression, anxiety, social support, and perceived stress, and for negative health behaviors (e.g., alcohol and drug abuse). A consideration of these other issues, in which marital satisfaction may contribute to mortality, would be an important topic for future research.

## 5. Conclusions

In conclusion, the results of this long-term follow-up study of 8945 tenured working males demonstrate that low marital satisfaction increased the long-term risk of stroke and all-cause mortality. This association was of a similar order of magnitude to those determined for established risk factors (smoking and leisure-time physical activity) for all-cause mortality. Assessing marital satisfaction and appraising the health benefits of marital education programs for young couples should therefore be implemented as part of health promotion strategies for the general population.

## Figures and Tables

**Table 1:** Baseline characteristics of the Israeli Ischemic Heart Disease (IIHD) study population across marital satisfaction groups (second examination, 1965).

Characteristic	Total Study Population	Category 1 (*n* = 3829)	Category 2 (*n* = 4052)	Category 3 (*n* = 741)	Category 4 (*n* = 323)	P of Trend
Age (y)	49.1 ± 6.7	49.5 ± 6.7	48.8 ± 6.6	49.1 ± 6.7	49.4 ± 6.8	*p* < 0.01
Systolic pressure (mmHg)	138 ± 22	139 ± 22	137 ± 21	136 ± 21	136 ± 20	0.01
Diastolic pressure (mmHg)	84 ± 11	87 ± 12	86 ± 12	85 ± 12	86 ± 11	*p* < 0.01
Total cholesterol (mg/dl)	208 ± 39	208 ± 39	207 ± 38	206 ± 38	208 ± 40	0.36
HDL-c (mg/dl)	40.8 ± 9.6	40.9 ± 9.5	40.7 ± 9.7	40.8 ± 9.5	40.0 ± 9.9	0.22
Diabetes (%)	4.6	4.9	4.2	4.0	6.2	0.19
Smoking (% at any time)	67.7					*p* < 0.01
Never smoking	32.3	32.1	33.3	31.1	24.3	
Quit smoking	16.8	17.4	16.9	14.3	15.1	
1–10 cigarettes p/day	15.2	14.5	15.3	16.5	18.0	
11–20 cigarettes p/day	16.3	17.1	15.6	15.8	16.7	
20+ cigarettes p/day	19.4	18.9	18.9	22.3	25.9	
BMI	25.9 ± 3.2	25.9 ± 3.3	25.9 ± 3.2	25.9 ± 3.2	25.8 ± 3.5	0.99
Leisure-time physical activity (%)						0.15
None		60.4	57.6	57.4	59.0	
Sporadic		13.7	15.7	15.8	14.7	
Light		18.1	18.7	18.1	18.3	
Intensive		7.8	8.0	8.7	8.0	
SES	2.6 ± 1.2	2.6 ± 1.3	2.6 ± 1.2	2.4 ± 1.1	1 ± 1.2	*p* < 0.01
Family problems	3.5 ± 2.4	2.7 ± 2.1	3.0 ± 2.2	4.3 ± 2.3	4.5 ± 2.3	*p* < 0.01

Marital satisfaction categories: marriage is perceived as 1–very successful, 2—successful, 3—not so successful, 4—unsuccessful.

**Table 2:** Adjusted hazard ratio for all-cause mortality by categories of marital satisfaction (1965).

Analysis		Stroke Mortality		All-Cause Mortality	
		HR	95% CI	HR	95% CI
Model 1					
	Category 2	1.11	0.93–1.32	0.99	0.94–1.14
	Category 3	1.16	0.86–1.57	1.04	0.94–1.15
	Category 4	1.75	1.21–2.53	1.23	1.07–1.40
Model 2					
	Category 2	1.18	0.99–1.41	1.02	0.97–1.08
	Category 3	1.3	0.96–1.76	1.08	0.98–1.20
	Category 4	1.95	1.34–2.82	1.26	1.10–1.44
Model 3					
	Category 2	1.15	0.95–1.38	1.02	0.96–1.08
	Category 3	1.11	0.80–1.55	1.06	0.95–1.18
	Category 4	1.94	1.41–2.90	1.21	1.04–1.41

HR = hazard ratio, CI = confidence interval. Marital satisfaction categories: marriage is perceived as 1—very successful, 2—successful, 3—not so successful, 4—unsuccessful. Model 1: Adjusted for age. 5736 died, including 595 stroke victims, out of 8945 subjects. Model 2: Adjusted for age, ischemic heart disease, diabetes mellitus, systolic blood pressure, smoking, BMI, leisure-time physical activity, and SES. Model 3: Model 2 further adjusted for serious or very serious family problems.

## Data Availability

The data presented in this study are available on request from Prof. Goldbourt, an author in this publication, who is legally responsible for the IIHD study database and has approved its use for the purpose of this study.
